# A Case of Doubtful Aneurism

**Published:** 1807-10

**Authors:** 


### *To the Editors of the Medical and Physical Journal.*

Gentlemen,

Did I not perceive that your Journal is open to the young and the anonymous, as well as to the titled veterans in practice, and that the cursory remark, the individual opinion, or the speculative suggestion, are not entirely excluded by the laboured essay or finished detail, I should not have appropriated a moment of your time to the perusal of these pages. But this consideration, aided by my natural *cacoethes scribendi,* acts so powerfully upon my will, at this moment, that the consequence is—that I must write a letter to the Editors of the Medical and Physical Journal. This determination, I will frankly confess, is not the result of a conviction, that any knowledge which I possess, were it all communicated, could either instruct the ignorant, or confirm the opinion wavering between some controverted points in practice. But I am no convert to the well-known maxim "Vir sapit qui pauca loquitur: on the contrary, I conceive that the man who delivers his opinion upon every subject, who conceals not any remark he has made, however trivial, is of infinitely more advantage to society and to science (be his attainments ever so small) than the phlegmatic sons of solemn taciturnity, who hug themselves in the belief that they are acquainted with something of which the world is ignorant, and hoard up with miserly care the precious treasure: a conduct, which, though it is sometimes the attendant of real knowledge, is generally the badge of ignorance and a narrow mind. In our profession, in particular, which can never be perfected by solitary study or abstract reasoning, but by the accumulation of experience, the disclosure of every fact or observation relative to practice (avoiding the repetition of what every one knows) must be of consequence; and can, *at the worst,* add but additional support to facts which cannot be too well known. With this disposition, I have resolved to communicate to you, at any time, whatever circumstance comes under my observation, which may in the smallest degree tend to the benefit of the profession; and I may add, that I have full opportunities of attending to a particular department of medicine, which is of the utmost importance, and which, notwithstanding the exertions of some late eminent writers, is not too well known; I mean Naval Practice. I hope you will not consider it as derogating from my credit, when I inform you that I am but a young practitioner.

 However

However, if one takes the trouble to observe and to think, any striking appearance, or any deviation from the laws that are laid down by the great oracles of physic, is more apt to make an impression on the young mind, than on the old practitioner, whose curiosity is blunted, and who has insensibly fallen into a routine of practice, fatal to exertion and improvement; an effect which we see produced *every day*.—So much for introduction.

From my short residence in my present situation I have seen little new, and I shall only detain you at present, by requiring the opinion of some of your Correspondents, on a case which, though perhaps not very singular or interesting to the medical world, is of the utmost importance to the suffering individual.

A seaman, of about forty years of age, of a robust habit and athletic form, about two weeks ago, complained of *pain of breast*. Upon examination, the temperature of his skin was found considerably above the natural standard, his pulse extremely full, and upwards of 90. The place he pointed to, seemed higher up than is commonly the case in pulmonic inflammation; and upon removing his clothes a small tumour was discovered just above the clavicle, where it joins with the sternum; it felt circumscribed, about the size of half a large hazle-nut, distinguishable to the eye only by its strong pulsation, and indeed so strong as if to elevate the pressed finger along with it upon the stroke of the pulse. It communicated a peculiar sensation to the finger, a kind of *hissing* feel, if such an expression could be used; or as if a large body of fluid were pressed upon a small orifice. Nothing more appeared in the case deserving attention. The anterior branch of the temporal artery on the right side (where the tumor is situated), gave a much less pulsation than that of the left, and any difference of force in the radial arteries at the wrists was imperceptible. *He attributes it to a bag of bread that was thrown on his head and forced it towards the left shoulder, about a month ago.* Is this an aneurism of the carotid artery? and how is it to be treated? The man was discharged to his duty, where he still continues, and complains of liitle uneasiness from his neck. His arterial system still continues in the same apparently increased action; but whether this be the effect of idiosyncrasy or disease, I cannot determine.

Yours, &c.

F. G.

His Majesty's Ship ——————.
*Plymouth, Sept.* 10, 1807.

